# Moral Distress and Austerity: An Avoidable Ethical Challenge in Healthcare

**DOI:** 10.1007/s10728-019-00376-8

**Published:** 2019-07-17

**Authors:** Georgina Morley, Jonathan Ives, Caroline Bradbury-Jones

**Affiliations:** 1 Department of Bioethics, Heart and Vascular Institute, Cleveland Clinic, Main Campus, 9500 Euclid Avenue, Cleveland, OH 44195 USA; 2 Centre for Ethics in Medicine, University of Bristol, Bristol, UK; 3 School of Nursing, University of Birmingham, Birmingham, UK

**Keywords:** Austerity, Moral distress, Bioethics, Nursing, Phenomenology, Empirical bioethics, Feminist empirical bioethics, Resilience, Moral resilience, Critical resilience

## Abstract

Austerity, by its very nature, imposes constraints by limiting the options for action available to us because certain courses of action are too costly or insufficiently cost effective. In the context of healthcare, the constraints imposed by austerity come in various forms; ranging from the availability of certain treatments being reduced or withdrawn completely, to reductions in staffing that mean healthcare professionals must ration the time they make available to each patient. As austerity has taken hold, across the United Kingdom and Europe, it is important to consider the wider effects of the constraints that it imposes in healthcare. Within this paper, we focus specifically on one theorised effect—moral distress. We differentiate between avoidable and unavoidable ethical challenges within healthcare and argue that austerity creates additional avoidable ethical problems that exacerbate clinicians’ moral distress. We suggest that moral resilience is a suitable response to clinician moral distress caused by unavoidable ethical challenges but additional responses are required to address those that are created due to austerity. We encourage clinicians to engage in critical resilience and activism to address problems created by austerity and we highlight the responsibility of institutions to support healthcare professionals in such challenging times.

## Introduction

Austerity, by its very nature, imposes constraints. It does so by limiting the options available to us because certain courses of action are too costly or insufficiently cost effective. In the context of healthcare, the constraints imposed by austerity come in various forms; ranging from the availability of certain treatments being reduced or withdrawn completely, to reductions in staffing that mean healthcare professionals (HCPs) must ration the time they make available to each patient. These constraints create and exacerbate ethical challenges in healthcare.

As austerity has taken hold across the United Kingdom (UK) and Europe, it is important to consider the wider effects that these constraints impose on healthcare. The effects are multifactorial, as evidenced by this special edition, but in this paper, we focus specifically on one theorised effect—moral distress (MD). In this paper, we draw on data gathered and analysed as part of a larger project exploring MD in UK nursing. The purpose of the original study was to explore the concept of MD as experienced by critical care nurses and the first author (GM) provided a reconceptualisation and redefinition of MD in her Ph.D. thesis. Consequently, much of our discussion originates from a nursing perspective, but the underlying ethical challenges are applicable to all HCPs. We are applying our analysis of the empirical data to the context of austerity and to the responses to austerity. In particular, to the response that healthcare staff should be more resilient to overcome the additional challenges that have arisen because of austerity measures and resource restrictions [70].

Ethical challenges created by austerity should, we argue, be considered avoidable because they are the product of contingent, rather than necessary, features of healthcare work. They create burdens and ethical challenges for HCPs that go beyond the inevitable ethical considerations of balancing harms and benefits of different treatment options, patient suffering and end-of-life decision-making. Ethical questions regarding withholding or withdrawing life-sustaining treatments (LST) are unavoidable and will likely always be a cause of moral distress (MD). Even with infinite resources, clinicians would still need to ask whether it is ethically permissible to provide or continue LST and balance this with quality of life considerations. As some scholars have suggested, MD in these situations could be considered a natural response to morally troubling situations [23, 45, 69], and no amount of resources could mitigate the nature of these decisions. This is not to say we should ignore the MD that occurs due to unavoidable ethical challenges but rather that avoidable and unavoidable ethical challenges require different responses. We will discuss these different responses towards the end of the paper.

We first outline the concept of MD before describing the larger study and presenting data that challenges the idea that MD is caused only by constraint. We explore the way that various examples of MD might be linked to austerity and consider how an appropriate response to MD and austerity might be interconnected through the concept of resilience, drawing on a distinction between moral resilience [54] and critical resilience [70]. We conclude that moral resilience is a useful response to the ‘unavoidable ethical challenges’ that arise in healthcare, but critical resilience is a more useful response towards the ‘avoidable ethical challenges’ that arise as result of austerity.

## The Moral Distress Debate

The term ‘moral distress’ was first coined by Jameton [25] who observed amongst nurses a tendency to feel distressed when they were forced to act, because of institutional constraints, in way that was contrary to their beliefs. Jameton consequently suggested that MD arises when “one knows the right thing to do, but institutional constraints make it nearly impossible to pursue the right course of action” (p. 6) [25]. Subsequent research, predominantly in the United States (US), but increasingly in Europe [11, 14, 47] has supported this conception of MD, showing that feelings of distress amongst HCPs are associated with being constrained in this way. Due to the presence and effects of austerity, it seems coherent to suggest there will follow an increase in the prevalence of MD amongst HCPs who will be forced to act in ways they feel are ethically sub-optimal. This will, subsequently, amplify the negative effects of austerity on patient care, given that MD is associated with increased rates of compassion fatigue, burnout and intention to leave the profession [22, 36, 46]. If we accept this, then the simple way to reduce MD in the HCP workforce is to abandon austerity, make more resources available, thereby removing constraints. However, our findings suggest that the removal of resource constraints will only mitigate the MD that is caused by avoidable ethical challenges (such as those caused by austerity) and will do nothing for clinicians experiencing MD because of unavoidable ethical challenges. We argue, similarly, that MD arising out of austerity cannot, and should not be responded to with simple calls for HCPs to become better at coping.

In recent years, a growing number of scholars have argued that MD should not be associated uniquely with constrained moral judgement, but should instead be understood more broadly [8, 19, 40]. Johnstone and Hutchison [27] argued that Jameton’s [25, 26] ‘narrow’ conception (as coined by Fourie [19]) perpetuates the assumption that nurses’ moral viewpoints are correct and justified, and that other members of the healthcare team are simply failing to recognize the correct moral action. They argue that this risks shutting down moral deliberation between HCPs and GM has argued that this prevents the formation of moral communities [37]. Indeed, our research provides empirical data to support the view that psychological distress which occurs in response to a variety of moral situations should be regarded as *moral* distress, as long as the causal criteria between the moral event and psychological distress are fulfilled. As such, we have posited the following definition of MD being a combination of:

(i) The experience of a moral event which could be any/combination of the following: moral tension, moral conflict, moral dilemma, moral uncertainty or moral constraint.
(ii) The experience of ‘psychological distress’ which is an umbrella term to capture a variety of negative emotions such as anger, frustration, guilt, regret, sadness/upset, powerlessness and symptoms associated with stress.
(iii) A direct causal relationship with (i) and (ii) [37] .^1^

Moral events were identified according to whether participants described or identified that significant moral events were at stake. Each moral event (listed above) was characterized and defined using the participants’ experiences and ethical theory. For example, moral conflicts and moral dilemmas were defined according to Tessman’s [64, 65] work because participants’ experiences reflected this conceptualization. Following Fourie [19], we suggest sub-categorising types of distress according to the moral cause so MD caused by a constraint is regarded ‘moral-constraint distress’, MD caused by a moral conflict is ‘moral-conflict distress’ and so forth.

## Methods

The overarching methodology for the larger project was feminist empirical bioethics (as coined by Scully) [57] because empirical data were combined with feminist ethical theory to inform conceptual development of MD and the normative recommendations for ways to respond. The project was feminist because of the focus on issues of voice, power, particularity and relationships [16, 41]. This methodological underpinning also allowed us to explore the issue of social justice which is central to the feminist approach and therefore GM was attentive to participants who wanted to frame their experiences within the larger political structures and climate.

Adopting a progressive reading of Heideggerian interpretive phenomenology, phenomenology was combined with feminist theory to create a feminist interpretive phenomenological approach [18, 61]. GM interviewed (*n *= 21) critical care nurses from two acute care NHS hospitals about their ethical experiences, how the experiences made them feel and how they conceptualised MD. Participants knew the study was about MD as this was necessary for informed consent, but the concept was not pre-defined. The interviews were open and non-directive so participants could lead the interview. The aim of the researcher in an interpretive phenomenological study is to immerse oneself in the ‘hermeneutic circle’, which means moving between the singular experiences of each participant to jointly shared experiences [31]. This required first coding the transcripts line-by-line to create individual themes, re-writing individual narratives to determine the prevalent moral events and then identifying the shared experiences that constituted generalised themes. Data were analysed according to Van Manen’s [75] interplay of six activities which involve investigating the phenomenon; immersing oneself in the data; reflecting on essential themes; describing the phenomenon by writing and rewriting; maintaining a strong orientation to the data and balancing the research context by considering the parts and whole. During data analysis, GM critically analysed the data to explore which experiences could be determined ‘moral’ in nature and whether they could be causally related to subsequent descriptions of negative feelings that constituted distress. The process was reflexive and GM maintained a reflexive research diary throughout the process which acts as an audit trail to enhance the trustworthiness of the process [51].

## Empirical Findings

In the following, we present data on UK nurses’ experiences of self-reported MD and an analysis that highlights the way in which nurses’ experiences of MD can be correlated to resource constraints. We argue that these resource constraints present avoidable ethical challenges that (predominantly) cause moral-constraint distress because HCPs were required to ration their time, provide suboptimal care and leave care undone.

## Austerity and Avoidable Ethical Challenges

Following the Great Recession in the US in 2007 and the knock-on effects across the world, numerous governments across Europe enforced austerity measures in the hope that a reduction in public spending and increased taxation would help to mitigate rising debt and prevent economic collapse [62]. The focus of this paper is upon the effects of austerity on nurses through the lens of moral distress. In this section, we provide the data in which participants described a number of resource challenges that created and exacerbated the moral events they encountered and subsequent experiences of MD. Throughout this section, we highlight the ways in which these avoidable ethical challenges can be correlated to austerity measures.

### Lack of Sufficient Staffing

Over the past two decades there has been a growing body of literature reporting the inability of nurses to provide care due to time and resource constraints—variously labelled as ‘unfinished care’, ‘implicit rationing’ ‘missed care’ [28] and most commonly in the UK as ‘care left undone’ [5, 6]. This research has sought to evidence the correlation between registered nurse to patient ratios, nurse education and patient mortality to emphasize the importance of safe staffing levels and nurse education for both mortality and nurse job satisfaction [1–4, 59]. However, there has been limited research exploring the association between nurse staffing, the political context and the occurrence of moral challenges [72]. It is widely believed that undergraduate and postgraduate nursing bursaries were cut because of austerity measures [7] which had the (predictable) knock-on effect of decreasing the number of qualified nurses [43] and causing nurses to leave the NHS due to unsatisfactory working conditions [44, 52, 67]. The decrease in nursing numbers and subsequent increase in inadequate staffing levels have forced nurses to effectively ration care. Despite growing evidence, mandated safe staffing levels remain aspirational in England and as Lawless et al. [32] argue, this seems to be largely due to the tension between safe staffing and funding constraints.

Our data supported this, for example in the passage below from Phoebe:

a lot of people had a really sad Christmas here because they were stretched beyond what they should be and there was a lot of anger on the unit… because they felt utterly and completely abandoned. There were no matrons… everyone else was sitting at home and one nurse, one of my team, was quoted as saying ‘how bad does it have to get for someone to come in and help us… I know there was a lot of anger because the staffing shouldn’t be that bad but I don’t know if I would call it moral distress because it’s more… I guess yeah because a lot of the nurses did say, ‘this isn’t safe’, you know? We’re having to keep patients sedated because we haven’t got enough staff to cover if they’re not sedated and that’s not right, so I guess if you dig a bit deeper it would be moral distress but I don’t think that’s the first thing that would come to people’s minds (Phoebe).

In Phoebe’s narrative, she describes how the nurses working in her unit were unable to fulfil their professional responsibilities to patients because of understaffing. This resulted in patients being sedated and ventilated for longer, and rehabilitation opportunities missed, both of which are important for decreasing mortality and reducing length of stay in the Intensive Care Unit (ICU) [24, 42, 79]. A recent report from the Royal College of Nursing (RCN) provides evidence from nurses reporting similar experiences to Phoebe’s [52]. However, the report does not highlight the ethical implications of these episodes of missed care. Phoebe is describing how lack of sufficient staffing thwarted the nurses’ ability to fulfil their ethical obligations to patients because they were constrained. The presence of moral-constraint distress is evidenced by Phoebe’s description of her resulting feelings of anger and frustration, which can be interpreted as causally related to being “utterly and completely abandoned” and thus forced to provide care that was not only suboptimal but also unsafe.

Indeed, it is well documented that insufficient staffing causes moral-constraint distress. Using the Moral Distress Scale and the Moral Distress Scale Revised in various countries, the item ‘Work with levels of nurse staffing that I consider unsafe’ has consistently been one of the top four items reported as causing the most frequent and intense moral-constraint distress [12, 30, 48, 50, 60]. The emergence of this theme in our findings is therefore unsurprising. Peter and Liaschenko [49] highlight the relational barriers that understaffing creates because of the emphasis on efficiency and increased workloads, both of which are constraints that prevent nurses from forming relationships with patients and inhibits their ability to provide holistic care.

Scott et al. [56] argue that the ethical implications of missed care have been relatively unexplored and very rightly re-frame understaffing as a form of implicit rationing. They highlight how despite the reduction in nursing staff, nurses are still expected to fulfill patients’ needs fully [56]. The UK is now one of the highest ranking countries in Europe to cut health expenditure (with a drop in growth behind only Greece, Ireland, Estonia and Slovak Republic) [74] but there seems to be an expectation that quality should not be affected [32]. In reality, the lack of funding in healthcare and insufficient staffing means that nurses must prioritise and ration their time which leads to increased incidences of missed care.

Indeed, England reported higher incidences of unfinished care than the European average, along with Ireland, Belgium, Germany and Greece (in ascending order) [28].

From Pheobe’s narrative, we see how resource rationing negatively impacts both patient care and nurse job satisfaction, because nurses are forced to compromise the quality of care they wish to provide. Over time, compromised or crushed ideals leads to disillusionment, ‘job hopping’ or a decision to leave the profession [34] and negative perception of one’s ethical environment is also correlated to higher levels of moral-constraint distress and intention to leave [13, 21]. In our study, half of the participants (*n *= 11) reported either leaving, intending to leave their role or the profession. They cited several reasons but issues related to feeling over-worked, forced to provide suboptimal care, stressed and morally distressed made up part of their rationale:

You know, the NHS is the most affordable, best system ever and we’re having it pillaged under our noses and it’s like abandoning the sinking ship, isn’t it? You know. We’ve lost a couple of shiny new Band 5 s to go and work in the private system for forty-five grand a year doing like nine to five outpatient assessment stuff. Be f***ing mad not to, wouldn’t you, really?… It’s like well, yeah, but she’s probably not gonna be exhausted and she’s probably gonna get to spend weekends with her children when she has them and afford a house and stuff… they haven’t even got kids and whatever and you can see them burnt out, dropping like flies but then still doing bank shifts and things. Like I can’t do bank shifts. I, I’d have to do something else. I couldn’t do, um, be in there more than I have to be in there ‘cos it’s just so emotionally and physically exhausting, because of all those things like if it was smooth and managed properly and you weren’t, like, waiting for ever for, you know, it’s like, I’m thinking you know if you need like an ENT input and your hospital doesn’t have ENT, well then you’re gonna be waiting like, however long it’s gonna take, five days, ten days for ENT input from another hospital to come. It’s just…ridiculous things in the system. So I can’t separate the morality from the management I think. I think that’s where those strings are being pulled and that’s…but those pulling those strings aren’t ever accountable for the outcomes, it seems to me. It’s the doctor that fucks up at the bedside by accidentally putting some sort of anabolic intrathecally because he was like pulled in the night and didn’t sort of…stop and think what he was doing (Holly).

### Lack of Sufficient Skill Mix

Participants perceived staffing problems as circular: junior nurses joined critical care but would feel unsupported in their new role due to lack of sufficient staffing and poor skill mix and end up leaving. Joyce, a senior nurse, discusses the challenges this created for her both personally and professionally. Personally, she felt fearful because she didn’t know who she would be working with from one day to the next, and professionally it was difficult to allocate junior nurses to patients they could care for safely and feel supported.

here turnover is so high, so many junior staff, so many people leave every week, like every two weeks there are new starters and we didn’t have an awful lot of senior staff and a lot of people who had the course done so even coming back it was just like who am I going to be working alongside, like how far are they down their training and sometimes the allocations… the person might only have been on the job a month in the side room and might not be able to give medications yet and you might not have a floater to go round and give support, that’s a bit challenging (Joyce).

Joyce seems to be describing three different problems: understaffing, lack of sufficient skill mix leading to difficulties allocating patients and thirdly, the effects of hospital design on resource allocation decisions. Joyce described feeling angry and frustrated because she felt the care she provided was being compromised as a result of external factors that constrained her ability to fulfil her ethical obligations—the very definition of moral-constraint distress.

As with lack of sufficient staffing, poor skill mix should, we argue, be seen as another form of rationing causing nurses to feel unable to provide not only holistic but *safe* care. Despite an increasing and ageing population, the UK government have continued to enforce austerity measures by cutting health expenditure [68]. These cuts have had a huge impact on the current nursing workforce and nurses are reporting working in high stress environments which are untenable and unsustainable [52]. This seems to be both deterring people from entering the profession and causing them to leave resulting in a workforce gap [43, 67]. Danielle discusses her perception that the nursing role is being increasingly extended and stretched, and therefore causing individuals to feel increasing stressed and dissatisfied:

…they’ve got to be looking at budgets and have we got too many agency staff on and… especially the nurse’s role, it’s been turned into so many other different things that it wasn’t initially that people’s resources and capabilities are stretched so thin that you’re being asked to do much more with less resources. And this has an impact on, on everything… (Danielle).

Numerous reports have echoed Danielle’s narrative and highlight poor working conditions, high levels of stress and pay restraint as factors causing nurses to leave the profession [43, 52, 66, 67]. Recent statistics from the Nursing and Midwifery Council show that more nurses and midwives are leaving the profession than joining it, and two of the most cited reasons for leaving the register were working conditions—specifically poor staffing levels and high workloads—and disillusionment with the quality of care that nurses reported feeling able to provide [44]. That nurses feel disillusioned highlights the potential for MD: they are providing suboptimal care which prevents them from fulfilling their moral responsibilities to patients. Similarly, other countries facing healthcare spending cuts because of austerity driven policies report staff demotivation, disillusionment and lower quality of care [9, 29, 33, 78].

Danielle highlights the potential effects of spending cuts beyond nursing, describing her perception that consultants may be reluctant to make ethical decisions about whether to withdraw LST because of lack of time and lack of support.

if you had a consultant speaking to you they would say, ‘Well, you know, I couldn’t see that family for three hours because I was dealing with x, y and z’… maybe they feel that they are in charge and they’re looked on to make all these decisions and they can’t make the decisions because they haven’t got the support behind them. And that’s why they pass it onto somebody else. I mean that would make a lot of sense (Danielle).

Participants discussed their perception that consultant decision-making was already highly variable and with added resource pressures decisions would be increasingly delayed. Variability in consultant decision-making has been labelled the ‘roster lottery’ by Wilkinson and Truog [77] and we suggest that resource constraints add an additional avoidable compounding factor which complicates end-of-life decision-making and increases inequity for patients. This causes moral-constraint distress for nurses who are then witness to these increased inequities and feel powerless to effect change.

The third issue raised by Joyce is hospital design, in particular the move to single patient rooms or side rooms [56]. Charge nurses are responsible for allocating care across their nursing units and some hospitals are now developing units that consist only of single-bed rooms. These present a substantial workforce planning challenge in ICU and charge nurses are responsible for making allocation decisions which have important moral implications. As Scott et al. [56], highlight, nurses are expected to make these decisions from their single perspective without an explicit and agreed decision-making framework in which care priorities are agreed.

Side rooms are seen as positive for patients because they provide increased privacy, reduced noise levels and a reduction in the transmission of hospital acquired infections [10, 73]. Although the connections between austerity and hospital design are not clearly reported, the development of single-bed only hospitals is another efficiency move that has been conducted with little attention to the effect on nursing staff. Participants reported being left in side rooms with critically ill patients for prolonged periods of time without assistance or support, leaving them feeling isolated and “trapped”. Participants described how this forced continued proximity seemed to magnify the emotions they experienced during moral events and increased their feelings of powerlessness and frustration associated with MD:

I just wanted to cry with the daughter and be like no I think you’re right but also I felt really trapped because physically I was in that side room and I couldn’t have anyone to be like ‘look come and look at him he’s dying; let’s stop this now…’ (Jenna).

This efficiency move, combined with staffing shortages, seemed to compound and exacerbate ethical challenges. In the previous quotation, Jenna discusses feeling trapped in the side room and unable to speak to colleagues about a patient who seemed to be dying. Jenna described experiencing moral-constraint distress because the team had decided to proceed with a time-limited trial of LST to see if the patient would improve. Jenna is describing the moment the patient’s daughter had realised these treatments were failing but Jenna was unable to escalate to the Consultant because she couldn’t leave the side room for assistance. Indeed, previous research exploring the effect of single-bed hospital design found that nursing staff felt teamwork was hindered and they had reduced ability to provide high-quality care [17, 35].

### Lack of Acute Care Beds

Participants discussed the broader ethical challenges that cuts in healthcare spending raised. In the following quotation, Rachel considers the fair distribution of resources and questions whether, as a society, we should be prioritising acute care and individuals who have lived a healthy lifestyle:

That’s five wards worth of patients who are waiting, they’re elderly - we know there’s a growing population of elderly- and nothing has been put in place to cope… that’s why the wards are full, that’s why the hospital’s chocker, that’s why we can’t get people in because they’re full of people waiting for social care and because it’s under a different umbrella… it’s obviously our tax money but it’s not NHS money, it’s social money… It’s just a big political mess and that’s why I think rather than spending money on people who are not prepared to look after themselves, why aren’t we looking after those people who have looked after themselves all the time and give them a decent end to their life… (Rachel).

Rachel raises important societal questions about public health considerations which need to be balanced with issues of social justice because of health inequalities. Nonetheless, it is interesting that Rachel has made this point. She seemed to express moral-uncertainty distress though frustration and anger as she questioned who was more deserving of her limited time. The increasing focus on spending inevitably raises biases that we all hold and which have the potential to impact care quality. In an ideal world, HCPs could be protected from the effects of extreme governmental policies so they can concentrate on providing equal and unbiased care.

Lawless et al. [32] highlight the government response to the Francis Inquiry, an inquiry into huge failings at Mid Staffordshire Hospital in which patients received suboptimal care. Many patients died due to a focus on cost savings above safety and a toxic culture:

This was a systemic failure of the most shocking kind, and a betrayal of the core values of the health service as set out in the NHS Constitution. We must never allow this to happen again…We will foster a climate of openness, where staff are supported to do the right thing and where we put people first at all times (pp. 5–6) [15].

Despite growing evidence that cuts to health and social care are causing increased mortality [76], increased staff dissatisfaction and loss of morale, the government are still failing to invest sufficiently in these areas thus mirroring the failings discovered in the Francis Report [20].

The lack of acute care beds also impacts the care nurses are able to provide. In the following quotation, Olivia articulates feelings of frustration and anger (moral-constraint distress) because she is forced to create new beds in areas that are not routinely staffed:

…they did a private patient and they wanted private patient heart valves done. There were no beds and I said if you want it done then you need to take out one of our delayed discharges that’s the only way we can create a bed, you know, and I said ‘no that’s the only way’ and they were, ‘well we’ll do them’ and I said ‘no, there are no beds’; ‘well we can open Recovery’; ‘no we can’t keep…, there are no nurses to keep opening these extra beds’. We put out to agencies, they don’t get filled, its unsafe, it’s not nice, I wouldn’t want my relative to be nursed in Recovery because there’s no Critical Care bed, they should be where they should be (Olivia).

## Responses to Moral Distress

The data presented above illustrate various ethical challenges associated with the experience of MD. Further, we have highlighted how some ethical challenges are integral to healthcare—such as whether to withdraw/withhold LST. These unavoidable ethical questions relating to patient suffering and quality of life predominantly raise questions that often do not feel so easy to answer. Subsequently, many participants in our study expressed moral-uncertainty distress because these ethical dilemmas could not be easily resolved. The ethical challenges brought about by a lack of resources which are highlighted in our analysis—which constrain action—are amplified by austerity.

In addition to the need for clinical ethics support to help manage the unavoidable ethical issues that arise in healthcare, resilience has also been mooted as an appropriate way to deal with MD—and this is at first glance plausible. Moral complexity and moral dilemmas are inevitable within healthcare, and feelings of moral failure^2^ and MD are also therefore inevitable [38]. It seems reasonable to propose that staff are supported to develop the skills to cope with the emotional distress that moral challenges provoke.

As with the concept of MD, resilience can be understood in different ways [80]. Two different conceptions of resilience that might be helpful are moral resilience and critical resilience. Developing the skills of moral resilience is thought to aid clinicians in choosing how they respond to ethical dilemmas so as to minimise personal suffering [53]. Rushton suggests that moral resilience can help individuals create meaning out of situations that cause cognitive dissonance and appear senseless, and to help individuals retain a sense of moral agency in a turbulent world [53, 80]. Sala Defilippis et al. [55] conceive of moral resilience as within an Aristotelian framework of virtues and vices, framing moral resilience as a virtue because the nurse would have the ability to remain open to compromise when faced with a situation in which they disagree on moral grounds.^3^ Indeed, this conceptualization seems to have utility for unavoidable ethical challenges. For example, if a nurse thinks it is morally wrong to stop ECMO for a patient with two young children because we should exhaust all options, but the patient’s husband informs the healthcare team that she would not want to continue living with the predicted quality of life the doctors describe. The nurse may experience moral-constraint distress because these values do not align with her own but this does not mean withdrawing ECMO is morally wrong. According to Sala Defilippis et al.’s [55] conception, the morally resilient nurse would be able to work through this moral challenge, have the ability to understand the differences in values and continue to care for the patient. To develop moral resilience, HCPs would need continuing clinical ethics education with particular focus upon how to work towards resolution and compromise [38].

However, as some commentators have argued—focusing on personal or moral resilience risks placing all the burden on the individual and absolves the institution of responsibility [70, 71]. In the context of austerity and cuts to resources, responses that only target individual responses are insufficient. Avoidable challenges created because of systemic and political austerity policies require a different kind of resilience. Traynor critiques the notion of personal resilience, arguing that it is “a term that supports the status quo” (p. 5), and that resilience teaches nurses how to “roll with the punches”, successfully subverting a huge workforce into believing if they learn how to overcome challenges on a personal level they can survive in healthcare [71]. According to this line of thought, once it is accepted that HCPs need to be resilient to cope with a particular kind of challenge, it is then expanded to cover all challenges, becoming a universal curative. Traynor suggests that HCPs adopt a different kind of resilience—‘critical resilience’—for the basis of activism and change [70, 71] to address avoidable challenges that arise from institutional decisions. To develop critical resilience, HCPs should critically engage with their environments to reorient complaint and criticism into critique regarding how external forces contribute to poor practices. In addition, HCPs could become politically active, this may include participating in direct action such as protests and rallies, writing letters to newspapers or connecting with elected officials [39].

Put simply, in responding appropriately to MD we need to consider the cause of distress which is likely to determine the most appropriate response. Some ethical challenges are an inevitable part of clinical practice to which developing personal coping strategies are indeed helpful. However, both moral and critical resilience still call upon the HCP to utilize inner resources, time and energy to continue functioning within the healthcare system. Ideally, there would and indeed we argue that there should be additional resources to help HCPs manage both avoidable and unavoidable ethical challenges and the types of moral distress these cause. We argue that institutions should develop mechanisms to determine the type and cause of MD to direct their responses. These mechanisms could be led by clinical ethics support services that are available to respond not only to clinical ethics dilemmas and conflicts but are also attentive to MD experienced by clinicians. Responses to unavoidable ethical challenges may consist of services available to engage with and listen to relevant stakeholder’s perspectives to provide ethically supportable recommendations. Whereas responses to avoidable ethical challenges would likely require much more of an advocacy role that would extend beyond the boundaries of the traditional ethics consultant model [58].

Healthcare institutions have a moral responsibility to create and sustain safe and healthy work environments, and as Scott et al. [56], state if they fall short then they should at minimum acknowledge this and support staff who bear this moral burden. Where MD results from austerity, it is not only healthcare institutions but also the political system that should be held responsible. Taylor [63] highlights work conducted in the US by the National Academy of Medicine and the American Nurses Association around recognising and increasing awareness of working conditions and the effects on clinicians’ health. It is time for the equivalent UK bodies to call attention to and advocate for change for HCPs in the NHS, and it is time for the government to listen.

## Footnotes

1 The causal relationship may be explained and fulfilled using the following causal criteria: (1) There is a feeling of either: other-regarding or self-directed empathy for the individual(s) involved in the moral event; and/or recognition and acceptance of a feeling of personal/professional responsibility to those involved in the moral event, including towards oneself. (2) The nurse has a proximate relationship to the moral event in time and space. (3) The nurse experiences a combination of emotions that may be regarded as falling within the umbrella emotion ‘distress’ following involvement in the moral event.

2 We understand and define ‘moral failure’ according to Tessman as the feeling of failure that can occur when one is faced with a moral dilemma and the moral agent perceives all the available options as violating a non-negotiable moral requirement.

3 The first author highlighted the utility of compromise as a response to moral distress in her Ph.D. thesis.
